# Nicotine in floral nectar pharmacologically influences bumblebee learning of floral features

**DOI:** 10.1038/s41598-017-01980-1

**Published:** 2017-05-16

**Authors:** D. Baracchi, A. Marples, A. J. Jenkins, A. R. Leitch, L. Chittka

**Affiliations:** 1 Queen Mary University of London, Department of Biological and Experimental Psychology, School of Biological and Chemical Sciences, Mile End Road, London, E1 4NS UK; 2 University College London, Department of Genetics, Evolution and Environment, Faculty of Life Sciences, Gower Street, London, WC1E 6BT UK; 3 Research Centre on Animal Cognition, Center for Integrative Biology, CNRS, University of Toulouse, 118 route de Narbonne, F-31062 Toulouse cedex 09, France

## Abstract

Many plants defend themselves against herbivores by chemical deterrents in their tissues and the presence of such substances in floral nectar means that pollinators often encounter them when foraging. The effect of such substances on the foraging behaviour of pollinators is poorly understood. Using artificial flowers in tightly-controlled laboratory settings, we examined the effects of the alkaloid nicotine on bumblebee foraging performance. We found that bumblebees confronted simultaneously with two equally rewarded nicotine-containing and nicotine-free flower types are deterred only by unnaturally high nicotine concentrations. This deterrence disappears or even turns into attraction at lower nectar-relevant concentrations. The alkaloid has profound effects on learning in a dose-dependent manner. At a high natural dose, bees learn the colour of a nicotine-containing flower type more swiftly than a flower type with the same caloric value but without nicotine. Furthermore, after experiencing flowers containing nicotine in any tested concentration, increasing numbers of bumblebees stay more faithful to these flowers, even if they become a suboptimal choice in terms of reward. These results demonstrate that alkaloids enhance pollinator flower constancy, opening new perspectives in co-evolutionary process between plants and pollinators.

## Introduction

Many plants defend themselves against herbivores by producing high concentrations of distasteful and toxics secondary metabolites, such as terpenes, phenols and alkaloids in their tissues^1^. Such substances are also frequently found in pollen and nectar, albeit in much lower concentrations than in most tissues^2, 3^. Many angiosperm plants depend on pollinators’ services for reproduction^4^ and lure pollinators using nutritious, sweet nectar provided in attractive flowers. The presence of feeding deterrents in nectars is therefore seemingly counterintuitive because it means that pollinators encounter them when visiting flowers^5^, but they may act to reduce nectar robbing and flower herbivory and manipulate frequencies of flower visitations^6, 7^. Pollinators have a prominent role in selecting nectar composition, possibly forcing a trade-off between herbivore defence and pollinator attraction in plants^8^.

Nectar phytochemicals can induce a variety of dose-depend adverse health effects on pollinators^9^. However, toxic effects often require much higher doses than those observed in natural nectars^9^. There is a growing body of literature showing a tendency towards enhanced feeding deterrence with increased alkaloid concentration^10–14^ but there is little evidence of complete deterrence by alkaloids in either honeybees or sunbirds^12, 13^, even at unnaturally high, potentially lethal doses^15^. Conversely, some nectar secondary metabolites can act as attractants themselves^16, 17^. Various alkaloids may have a prophylactic or therapeutic role in reducing pathogen load in pollinators^18–20^ and bumblebees may actively search for nicotine-enriched nectar in order to keep pathogens at bay^19^.

Remarkably, there is also recent evidence that some alkaloids may be able to manipulate the behaviour of pollinators pharmacologically, leading to enhanced pollination services^21, 22^. Alkaloids at high concentrations are known to be potent neurobiological agents, able to interfere with neural pathways including sensory and motor functions, cognitive abilities and learning processes in both vertebrates and invertebrates^23–26^. Similar to effects observed in mammals^23^, low, non-lethal concentrations of those molecules can act as psychostimulants in invertebrates^22^. When ingested they can lead to enhanced excitation of the reward system in the brain and positive appetitive reinforcement, altering their behaviour and eliciting a preference for nectars containing these chemicals^22, 27, 28^. During proboscis extension reflex (PER) conditioning, harnessed honeybees that were exposed to caffeine, which is a common nectar alkaloid naturally produced by *Citrus* and *Coffea* plants, performed three time better in both learning and memory retention tests than bees just rewarded with plain sucrose^22^.

In the present work, we studied the effects of the concentration of dietary nicotine, a natural alkaloid found in the nectar of several bee-pollinated plants, on the foraging behaviour of the bumblebee *Bombus terrestris audax*. Using flight arenas and artificial flowers rewarded with sucrose solution that was either laced with nicotine or nicotine-free, we designed two sets of experiments to test whether bumblebees display a preference for nicotine-laced nectar and whether this alkaloid is able to influence associative learning in bees.

## Results

**Figure 1:**
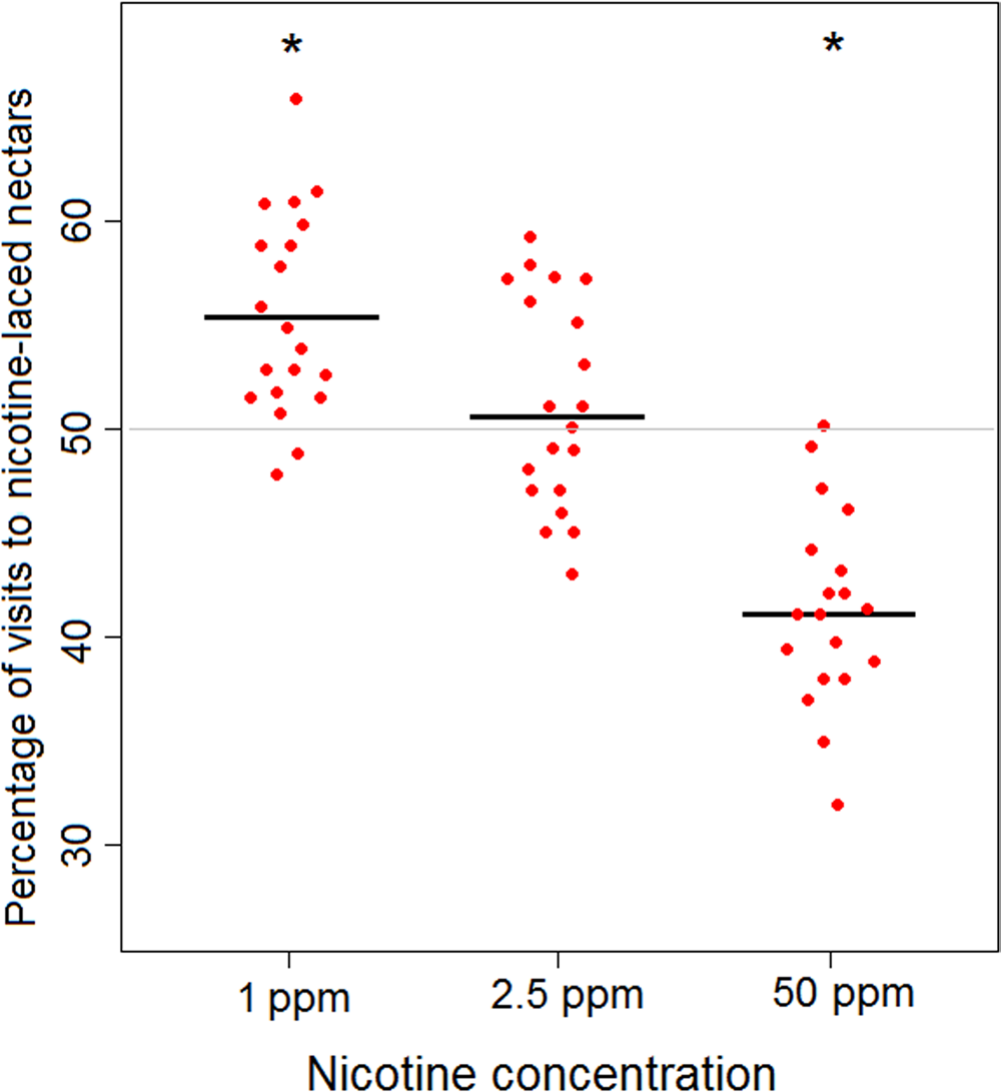
Bumblebee preference for nicotine-enriched nectars. Individual preferences (mean value of 100 choices) of bees (*n* = 60, 20 each nicotine concentration) for the flower type associated with nicotine-laced sucrose solution during a foraging choice test. Bees are deterred only by unnaturally high nicotine concentrations (50-ppm). This deterrence disappears or turns into attraction at lower nectar-relevant concentrations (1 and 2.5-ppm). Red dots represent individual bees, while the black bars indicate the mean value for each group. The grey line indicates random choice level. *P = 0.001.

In a first experiment, we tested whether bumblebees display a preference for nicotine-enriched nectars. Individual bumblebees (*n* = 60) were allowed to forage on an array of two flower types containing the same reward (30% sucrose solution), but differing in colour and the fact that one type contained nicotine in the sucrose solution. Three different concentrations of nicotine, two in the natural range (1-ppm and 2.5-ppm) and one above it (50-ppm) were used in three independent foraging tests. Bees were tested individually and one hundred consecutive choices were recorded. When compared with the expectation of a random preference, the low (1-ppm) concentration was attractive for bumblebees (One-sample t test, t = 5.3, df = 19, *P* = 0.001, mean ± SEM, 55.65% ± 1.1 of choices), whereas the high concentration (50-ppm) was repellent (t = −6.1, df = 19, *P* = 0.001, 41.35% ± 1.4 of choices). The intermediate (high natural) concentration (2.5-ppm) of the alkaloid did not affect bee foraging preference: choices in this case did not differ from random expectation (t = 0.96, df = 19, *P* = 0.34, 51.05% ± 1.1 of choice), (Fig. 1). Thus, when bumblebees were confronted simultaneously with two equally rewarded nicotine-containing and nicotine-free flower types, they were deterred only by unnatural high concentrations of the alkaloid. This deterrence disappeared or turned into attraction at lower natural doses.

**Figure 2:**
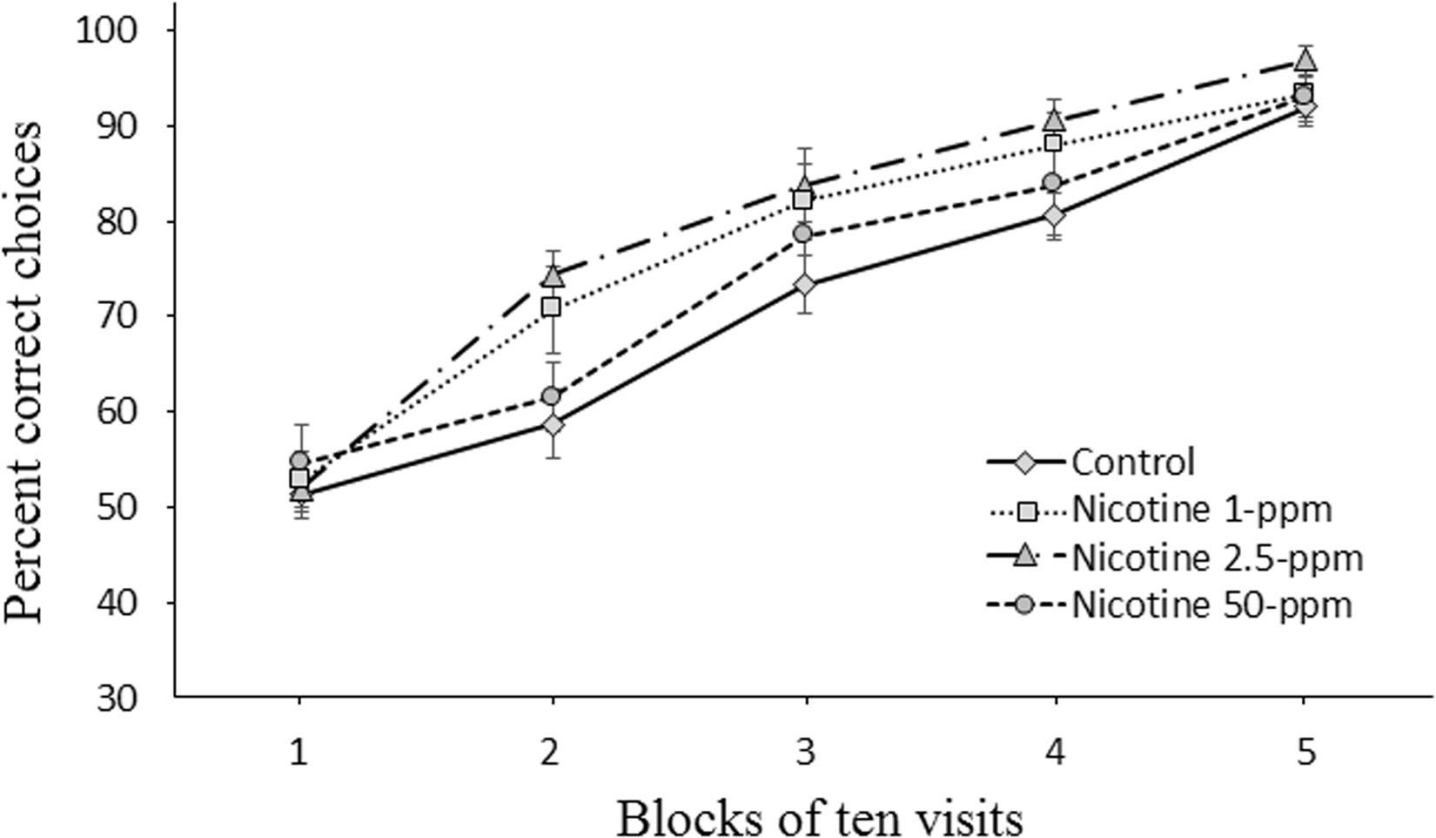
Nicotine-laced nectar enhances the forward learning performance of bumblebees during foraging. Learning curve (mean choice accuracy ± SEM) across 50 visits of bees exposed to 1-ppm (*n* = 15), 2.5-ppm (*n* = 16) and 50-ppm (*n* = 14) nicotine and not exposed (*n* = 15). Individual bees were allowed to forage on two flower types, one unrewarding (water only) and one rewarding (sucrose solution only (controls) or sucrose solution laced with nicotine). After a rewarding flower had been visited for the first time by a bee, 50 consecutive choices were recorded. Each learning curve was generated from the mean choice accuracies (percentage correct choices made) for every consecutive block of 10 choices. Overall, nicotine at 2.5-ppm significantly enhanced the speed of learning (*P* = 0.015) while the other two tested doses did not (1-ppm: *P* = 0.1; 50-ppm: *P* = 0.26).

A second experiment was designed to test whether the alkaloid is able to influence associative learning in bees. In this second experiment, naïve bumblebees (*n* = 60) were presented with an array of two types of flowers, either unrewarding (water only) or rewarding in independent tests, the reward being sucrose solution only (control), or sucrose solution plus 1-ppm nicotine, or sucrose solution plus 2.5-ppm nicotine, or sucrose solution plus 50-ppm nicotine (1-ppm and 2.5-ppm nicotine occur in natural range and 50-ppm above it^12, 15, 29^). We found that the presence of the alkaloid in nectar significantly boosts associative learning performance of bees, enhancing flower constancy once a flower type has been memorised. Indeed, over the foraging test, all the groups improved their performance showing that they learned to visits rewarding flowers and to avoid the unrewarding ones (GLMM, *trial*: χ^2^ = 299.4, df = 1, *P* < 0.0001). Yet, the mean choice accuracy of bees exposed to the nicotine + sucrose solution was greater than that of the control bees (exposed to sucrose solution only) across the entire forward learning phase (GLMM, *treatment*, χ^2^ = 8.39, df = 3, *P* < 0.038, Fig. 2). However, the effect was dose dependent and only the high natural concentration of nicotine (2.5-ppm) significantly enhanced the rate of learning through the test, while the other two tested doses did not (GLMM, Dunn’s test, 2.5-ppm vs control: *P* = 0.015; 1-ppm vs control: *P* = 0.10; 50-ppm vs control: *P* = 0.26, Fig. 2). By calculating the mean choice accuracies (percentage correct choices made) for every consecutive block of 10 choices for each group of bees, we found that, while at the beginning of foraging, bees of all groups had no preference for any flower colour (One-Way ANOVA, F = 0.23, *P* = 0.88, percentage correct choices (mean ± SEM): control 51.3 ± 2.6; 1-ppm 52.7 ± 3.2; 2.5-ppm 51.9 ± 1.9; 50-ppm 54.6 ± 4.2), bees exposed to 2.5-ppm nicotine-laced nectar performed better than control bees by the first 20 choices (Holm-Bonferroni corr., *t* = −3.65, df = 29, *P* = 0.003) and 40 choices (*t* = −3.19, df = 29, *P* = 0.009). Other tested concentrations did not influence bees preference at 20 choices (1-ppm: *P* = 0.09; 50-ppm: *P* = 0.56), 30 choices (1-ppm: *P* = 0.1; 50-ppm: *P* = 0.3) and 40 choices (1-ppm: *P* = 0.17; 50-ppm: *P* = 0.59). After 50 visits, choice accuracy approached saturation in all experimental and control groups (One-Way ANOVA, F = 1.01, *P* = 0.40, percentage correct choices (mean ± SEM): control 92.0 ± 2; 1-ppm 93.3 ± 2.8; 2.5-ppm 96.9 ± 1.5; 50-ppm 93.1 ± 2.1).

**Figure 3:**
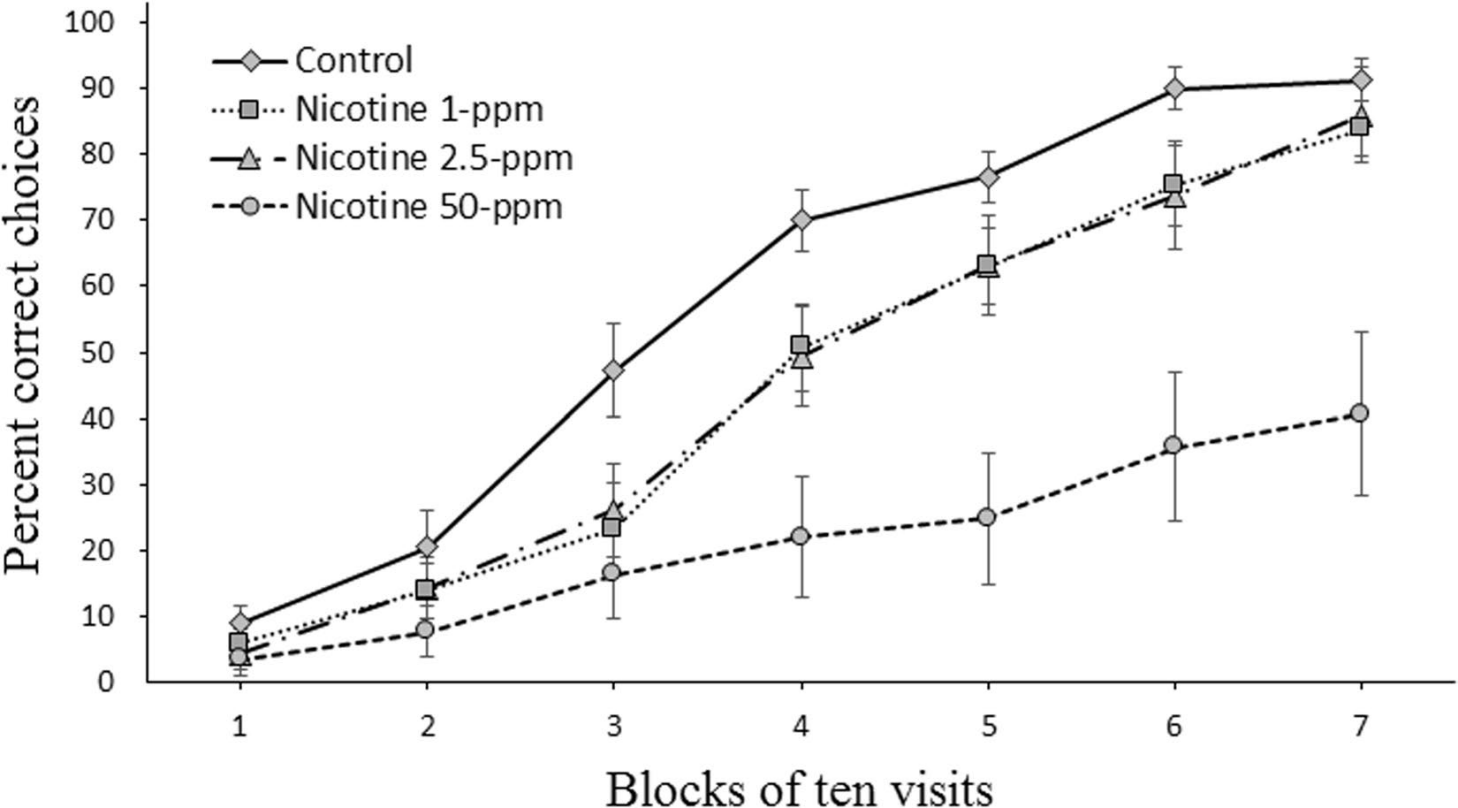
Nicotine previously encountered in nectar prompts bumblebees to make suboptimal choices in terms of reward. Reversal learning curve (mean choice accuracy ± SEM). After the forward learning phase, each bee underwent an individual reversal learning procedure in which the rewarding and unrewarding flower colours were switched and the rewarding flower for all groups provided sucrose solution (without nicotine). For each bee (*n* = 60) 70 consecutive visits were recorded. Learning curve for each group was generated from the mean choice accuracies (percentage correct choices made) for every consecutive block of 10 choices. Overall nicotine at 50-ppm strongly reduced the speed of learning (*P* < 0.001). A poorer performance than controls, yet not statistically significant, was also observed in bees previously exposed to the other two tested doses (1-ppm: *P* = 0.15, 2.5-ppm: *P* = 0.31).

In order to evaluate whether nicotine previously encountered in nectars prompts bumblebees to make suboptimal choices in terms of reward, we conducted a reversal learning test. Here bees which were previously allowed to forage on two flower types, one unrewarding and one rewarding sucrose solution (with or without nicotine at one of three dosages, see above) were immediately presented with the rewarding and unrewarding flower colours swapped. In this reversal learning test, all the rewarding flowers provided only sucrose solution. At the beginning of this test (i.e. the first block of 10 choices) bees of all groups had the same low foraging accuracy (One-Way ANOVA, F = 1.08, *P* = 0.36, percentage correct choices (mean ± SEM): control, 9.0 ± 2.8; 1-ppm, 6.0 ± 2.4; 2.5-ppm, 4.4 ± 2.0; 50-ppm, 3.6 ± 2.4, Fig. 3). Over the course of the test, bumblebees of all groups updated their information and improved their performance (GLMM, *trial*: χ^2^ = 879.8, df = 1, *P* < 0.0001, Fig. 3). However, the rate of learning differed between the treatment types. Bees previously exposed to nicotine were more likely to stay faithful to the previously rewarding flower type even though it had become the suboptimal choice in terms of reward (GLMM, *treatment*: χ^2^ = 31.59, df = 3, *P* < 0.0001, Fig. 3). The nicotine previously experienced in the forward learning phase (Fig. 2), now negatively affected the reversal learning in a dose dependent manner (Fig. 3). This time, the strongest detrimental effect on learning was induced by the highest nicotine dose (i.e. 50-ppm) (GLMM, Dunn’s test, *P* < 0.001), which made bumblebees overall more faithful to unrewarding flowers. Accordingly, the bees previously exposed to 50-ppm nicotine showed a worse performance at 30 (Holm-Bonferroni corr., *t* = 3.1, df = 26, *P* = 0.012), 40 (*t* = 4.35, df = 26, *P* < 0.003), 50 (*t* = 4.72, df = 15.6, *P* < 0.001), 60 (*t* = 4.96, df = 13.96, *P* < 0.001) and 70 choices (*t* = 4.31, df = 13.6, *P* = 0.003) than control bees (Fig. 3). Over the entire test, a poorer mean performance in term of accuracy than controls, yet not statistically significant, was shown by bees previously exposed to both 1 and 2.5-ppm (GLMM, Dunn’s test, 1-ppm vs control: *P* = 0.15, 2.5-ppm vs control: *P* = 0.31, Fig. 3).

**Figure 4:**
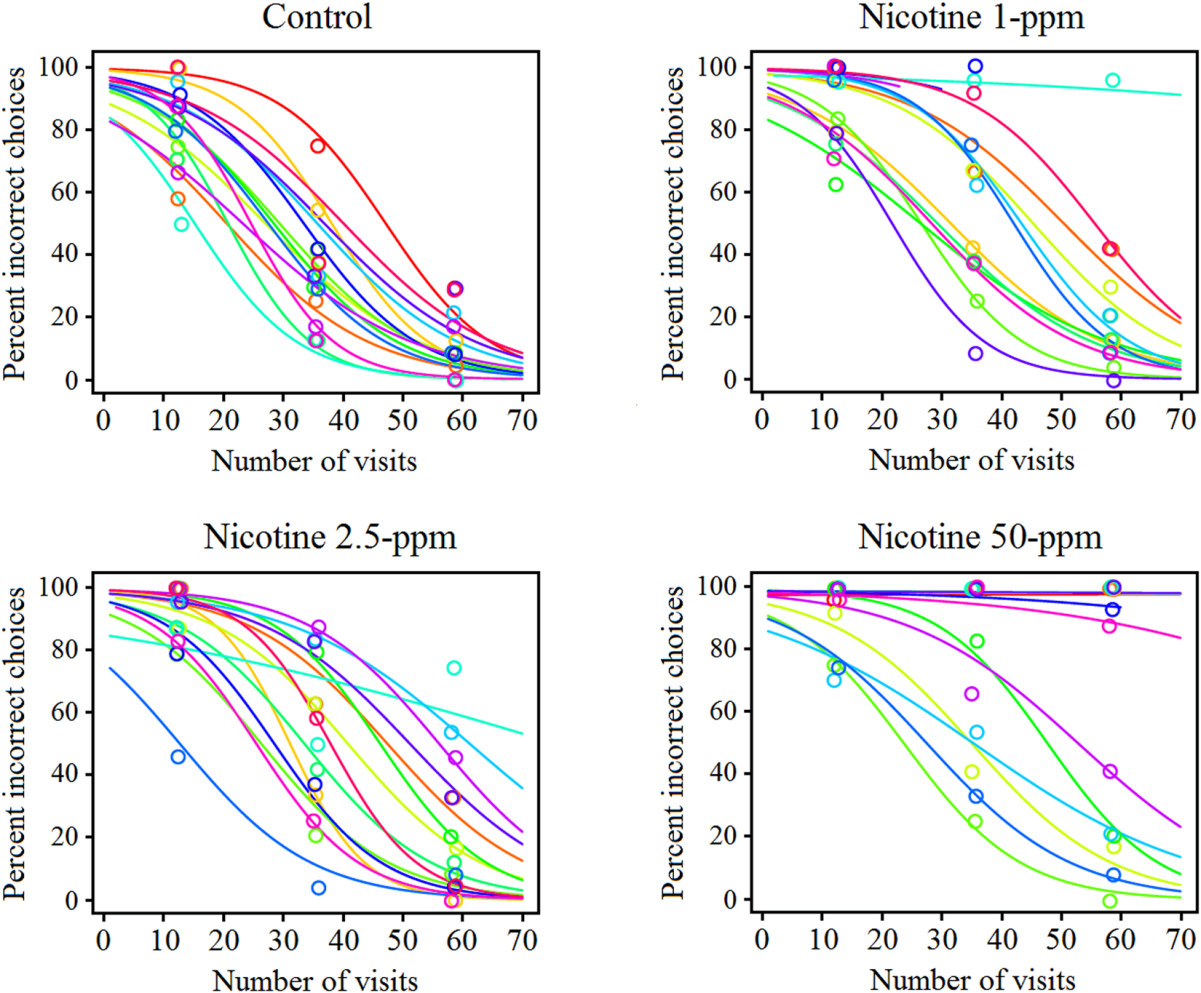
Individual bee performance in the reversal learning test. Plot of individual bee performance (*n* = 60) in the reversal learning test in which the rewarding and unrewarding flower colours were switched with respect to the forward learning phase, and the rewarding flower for all groups provided sucrose solution (without nicotine). For each bee (*n* = 60) 70 consecutive visits were recorded (*x*-axis). Different colours represent different individual bees. Circles represent raw data of bee choices that were pooled to 3 large bins of sequential choices for clarity. Curves were fitted to the 70 visits completed by individual bees. The plots reveal the large variation among individual bees’ responses. Some bees were unaffected by nicotine but an increasing number of bees were very slow or completely failed to revert with increasing nicotine concentration encountred in the forward learning phase.

A plot of individual bee performance in this reversal learning test revealed additional features of bee foraging accuracy (Fig. 4), these are: (1) some bees from each of the three experimental groups (1-ppm, 2.5-ppm and 50-ppm in associative learning phase) appeared to be unaffected by nicotine, even when previously exposed to the highest concentrations. Conversely (2), an increasing number of bees had reduced reversal learning accuracy with increasing nicotine concentration in the forward associative phase of the experiment. In addition (3), the relative accuracy of the bees changed little over the course of the experiment within all groups, that is, bees whose performance started well in the early phases of reversal learning, ended well too, and *vice versa*. The differences in term of accuracy observed in the reversal learning test were not due to differences in bee performance after the forward learning phase, as in that test, after 50 visits, all the groups had performed equally well (*P* = 0.40, see above). Similarly, bees of all groups had the same low foraging accuracy (*P* = 0.36, see above) in the first block of 10 choices in the reversal learning test (Fig. 3).

## Discussion

Our results suggest that the alkaloid nicotine either in concentrations within or above the natural range^12^ influences bumblebee flower preference via enhanced memory for floral traits and a reduced ability to reverse learn when the trait became sub-optimal. We also observed considerable variation between individual bees in terms of their response to nicotine, perhaps because of individual differences in susceptibility to the alkaloid, or other psychological traits^30^.

A positive impact on learning performance in bees has also been found in other nectar alkaloids such as caffeine, which helps bees to remember a learned floral scent three times longer than normal^22^. The identification of the mechanism of action of nicotine remains to be elucidated, but nicotine is an agonist of nicotinic acetylcholine receptors (nAChrs) par excellence. Thus, we speculate that this alkaloid, like caffeine, may act as a psychoactive drug^27^ by modulating the activity of cholinergic neurons in insect brains^23^. Acetylcholine is the primary excitatory neurotransmitter in the insect brain^23^ and cholinergic pathways are strongly implicated in many sensory functions, learning, memory and addiction^22, 23, 31^. After entering the brain via olfactory, tactile or post-gustatory pathways, the nicotine encountered by bumblebees may increase the rate of firing of neurons ﻿receiving acetylcholine at their input synapses﻿, leading to positive reinforcement of the flower-reward association^23, 32, 33^. This hypothesis is supported by recent evidence that some agonists of nACh receptors, chemically very similar to nicotine, such as the neonicotinoids imidacloprid and thiamethoxam, act to attract honeybees and bumblebees to sucrose solutions that have been laced with them^28^. Moreover the role of nicotinic receptors in memory formation in bees has been highlighted by other studies, in which nicotinic antagonist mecamylamine and the neonicotinoid imidacloprid are both shown to impair honeybee learning capabilities^31, 34, 35^. Similarly, the neonicotinoid clothianidin has been shown to impair learning in honeybees and to induce a faster learning in *Nosema*-infected bumblebees^36^. A second possible explanation for our findings comes from the evidence that nicotine lowers sucrose sensitivity threshold in honey bees increasing the subjective perception of reward quality^37^, which in pollinators is tightly linked to learning performance^38^. Thus, nicotine may also modulate the motivational state of pollinators by acting on distinct functional modules for reward learning like “wanting” and “liking”^39^.

Behavioural evidence from several studies suggests that bees can detect nicotine in floral nectar^10, 14–17, 19, 28^. We found that bumblebee preference for this alkaloid depends mainly on its concentration encountered in nectar. Bumblebees seem to be deterred only by unnaturally high concentrations of the alkaloid, while this deterrence is no longer evident at the high natural concentration, or it reverses to attraction at even lower concentrations. This finding, together with the recent evidence that bees prefer foods containing neonicotinoid pesticides^28^ suggest that pollinators might disproportionately visit plants treated with neonicotinoids. The volatile nature of this alkaloid^40^ suggests that it may contribute to the activation of olfactory sensory pathways. However, in honeybees at least, receptor cells for bitter substances have not been identified in the antennal tip so far^41^. Conversely, in both bumblebees and honeybees, a stimulation with nicotine dissolved in water elicited firing activity of several neurons located in the galeal sensilla, indicating that these pollinators have nicotinic gustatory receptors in their mouthparts^28^. This may explain how bumblebees detect this alkaloid in artificial nectars.

Nicotine occurrence in nectar is known to manipulate pollinator activity and enhance plant fitness^6, 7^. The results presented here show an additional dynamic in the manipulation of pollinators, whereby nicotine in nectar provides memorable signals so that plants of the same species are preferentially targeted by the same individual insects^42^. Such a response to nicotine in nectar may not apply to hummingbirds, since experiments with artificial flowers suggest that *Archilochus alexandri* ﻿only tolerates nicotine in sugar solutions when the birds had no other foraging choices, and prefer nicotine-free solutions given the choice^6^.

Bees have innate flower signal preferences that can be altered quickly depending on flower rewards, and bee visitation patterns become highly consistent once learned. Our study supports the idea that nectar alkaloids may increase pollinator constancy^21, 22, 24^. An enhanced memory of a rewarding flower type is likely to increase the rate of visitation to that plant species as bees are more inclined to visit flowers they have already learned to be rewarding than novel flower types^43^. Plants can have a selective advantage with enhanced outbreeding and reduce selfing, and it is likely that there are trade-offs between reproductive costs and reproductive success. The partial deterrence of pollinators by natural concentrations of nectar alkaloids may serve to increase the number of flower visitations by accelerating the rate at which they move from flower to flower^2, 39^, therefore increasing the rate of pollen transfer between flowers and promoting a higher rate of outcrossing^6, 44^.

In conclusion, pollinators might indirectly drive selection toward optimal concentrations of secondary metabolites while plants might pharmacologically manipulate pollinator behaviour, improving their own reproductive success. Such findings are unlikely to be restricted to caffeine and nicotine; other psychoactive compounds known from plants also have the potential to manipulate pollinator behaviour.

## Methods

### Animals and experimental set up

*Bombus terrestris audax* workers (provided by Koppert B. V., The Netherlands) were used in the experiments. Tests were conducted in the lab under standardized light (12:12, high frequency fluorescent lighting [(TMS 24F) lamp with HF-B 236 TLD (4.3 Khz) ballasts, Phillips, Netherlands fitted with Activa daylight fluorescent tubes, Osram]) and temperature (20 ± 2 °C) conditions. Before and after experiments colonies were provided *ad libitum* with pollen (Koppert B.V., The Netherlands) and sucrose solution 30% (w/w). All tested workers were uniquely marked with Opalith tags (Opalithplättchen, Warnholz & Bienenvoigt, Germany) for individual identification. Across all experiment, sucrose solution was at 30% (w/w) while nicotine ((-)-Nicotine hemisulphate salt (≥95% (TLC), ~40% (w/v) in H_2_O (based on free base); N1019 Sigma) was at 1, 2.5 or 50-ppm in sucrose solution 30% (w/w). Pollinators encounter nicotine in floral nectar of *Nicotiana* and *Tilia* species (the former being native to South America and naturalised worldwide by humans and the latter being native in most of the temperate Northern Hemisphere) at variable concentrations between 0.1 ng/μl and 3 ng/μl^12, 15, 29^.

### General procedure

Each colony was housed in a wooden box (28 × 20 × 11 cm) connected to a flight arena (120 × 100 × 35 cm) covered by a transparent UV-transmitting Plexiglas lid by a gated Plexiglas tunnel (25 cm length; 3.5 × 3.5 cm in cross-section). Several shutters along the length of the tunnel allowed us to control the traffic of the foraging bees between the nest box and the flight arena^45^. Artificial flowers were constructed from 24 × 24 mm transparent Perspex chips (Perspex^®^ Neutral) placed on top of clear glass cylinders to raise them above the green floor of the arena. During open foraging, these artificial clear flowers were organized in an array of 12 equidistant feeders and were all rewarding with a 15 µl droplet of sucrose solution. Flowers were immediately refilled after the bee moved to a different one by means of an electronic dispenser pipette (HandyStep electronic, BrandTech Scientific, Inc., Essex, CT, USA). All bees selected for the experiments were already actively foraging on the clear flowers but were naïve to both experimental flower colours and to encountering nicotine-enhanced nectar during foraging.

### Preference test for nicotine-laced nectar

To assess whether *B*. *terrestris* has a preference for nicotine-laced sucrose solution, we performed an experiment where bees were simultaneously presented with two different types of flowers (one laced with nicotine and the other nicotine-free).

Bees were trained to forage on 12 square transparent plastic flowers of 24 × 24 mm (Perspex^®^ Neutral) arranged in a rectangular grid consisting of 4 × 3 equidistant feeders to familiarize them with the artificial flowers. Motivated foragers were then tested in the same arena where the transparent plastic flowers were replaced with six blue (Perspex® Blue 272) and six purple (Perspex® Purple 4415) flowers associated with a reward (either 15 µl of plain sucrose solution or 15 µl of nicotine-laced sucrose solution) and alternatively arranged. Three different concentrations of nicotine, two in the natural range (1-ppm, 2.5-ppm) and one above it (50-ppm) were used in three independent tests carried out with three different groups of bees. During the tests, bees had to associate one colour with a reward (either plain sucrose solution or nicotine laced sucrose solution) and to make 100 consecutive choices. During the test, flowers were refilled so that bees never encountered an empty flower apart from the last one visited. Half of the bees were presented with purple flowers rewarded with plain sucrose solution and blue flowers rewarded with nicotine-laced sucrose solution, while the other half of the bees were presented with the opposite scenario. Twenty bees were tested individually at each concentration (*n* = 60 bees in total). Each bee was tested only once. Bees were considered to have chosen a flower only when they landed on it. New flowers were provided for each bee before individual testing to prevent possible use of scent marks as reward predictors^46^.

### Learning test

To test whether nicotine-laced nectar enhances the associative learning performance (speed of learning) during foraging, we subjected individual bees to a forward learning test and a subsequent reversal learning test. During the initial learning phase, unrewarding artificial blue (Perspex® Blue 272) flowers contained 15 μl of water for both control and experimental bees, while rewarding artificial purple (Perspex® Purple 4415) flowers contained either 15 μl of plain sucrose solution (control group) or 15 µl of nicotine-laced sucrose solution (experimental group). Choosing a rewarding flower was regarded as a ‘correct choice’, while choosing an unrewarding flower was considered an ‘error’. After a rewarding artificial flower had been visited for the first time by a bee, we started recording the number of correct choices. Also in this case three different concentrations of nicotine (1-ppm, 2.5-ppm and 50-ppm) were used in three independent tests. A total of 60 bees (*n* = 15, controls; *n* = 16, 2.5-ppm nicotine; *n* = 15, 1-ppm nicotine; *n* = 14, 50-ppm nicotine) were tested individually and 50 consecutive choices were recorded for each bee.

In order to test whether nicotine previously encountered in nectars prompts bumblebees to make suboptimal choices in terms of reward, soon after the forward learning phase (unrewarding flower providing only water or rewarding flower with sucrose solution, or sucrose solution plus nicotine at one of 3 doses (see above)), each bee underwent an individual reversal learning procedure in which the rewarding and unrewarding flower colours were switched and the rewarding flower for both groups provided sucrose solution (without nicotine). In this reversal learning phase, 70 consecutive choices were recorded for each bee. During both the forward learning and reversal learning phase, 6 blue and 6 purple flowers were alternatively arranged in a rectangular grid (3 × 4) of 12 equidistant flowers. The two colours were evenly interspersed and were either unrewarding or rewarding. The position of colours in the array was switched between each bout made by an individual bee to account for any positional bias. Flowers were cleaned between sequential bouts of the same bee, preventing the possible use of scent marks as predictors of as reward^46^.

### Data analysis and statistics

A two-tailed One-sample t-test was used to test for any bumblebee preference for nicotine-laced nectars in the preference test. Precisely, for each nicotine concentration we tested the observed sample against the hypothetical values of 0.5 which indicates the lack for any preference (i.e. a random decision take by foraging bumblebees).

Learning curves of the forward and reversal learning phase were analysed using ANOVA. Two independent models were carried out for the two learning phases. Bees’ choices (correct/incorrect) were examined using generalized linear mixed models (GLMMs) with a binomial error structure - logit-link function -, *glmer* function of R package *lme4* ^47^. In the models ‘*bee choice*’ was entered as dependent variable, “*treatment*” as fixed factor and ‘*trial*’ (i.e. the sequence of 50 and 70 visits over the forward and reversal learning phase, respectively) as covariate. Individuals﻿﻿’ identity﻿ (*IDs*) was considered as a random factor in order to allow for repeated measurements. We retained the significant model with the highest explanatory power (i.e. the lowest AIC value). In both selected models for the forward learning phase and the reversal learning phase the interaction term “treatment*trial” was not included. We used Dunnett’s post-hoc tests to detect differences between the different groups (*glht* function from R package *multcomp* ^48^). Finally we calculated the mean choice accuracies (percentage correct choices made) for every consecutive block of 10 choices for both the control and treatment groups^49^ and compared them using One-Way ANOVA or t-tests and Holm-Bonferroni correction for multiple comparisons (all the *P* values reported in the text are corrected). Statistical analyses were performed with R 3.2.3 (R Development Core Team, 2016).
